# A mesoporous non-precious metal boride system: synthesis of mesoporous cobalt boride by strictly controlled chemical reduction†

**DOI:** 10.1039/c9sc04498a

**Published:** 2019-11-15

**Authors:** Bo Jiang, Hui Song, Yunqing Kang, Shengyao Wang, Qi Wang, Xin Zhou, Kenya Kani, Yanna Guo, Jinhua Ye, Hexing Li, Yoshio Sakka, Joel Henzie, Yamauchi Yusuke

**Affiliations:** World Premier International (WPI) Research Center for Materials Nanoarchitectonics (MANA), National Institute for Materials Science (NIMS) 1-1 Namiki Tsukuba Ibaraki 305-0044 Japan HENZIE.Joeladam@nims.go.jp; Research Center for Functional Materials, National Institute for Materials Science (NIMS) 1-2-1 Sengen Tsukuba Ibaraki 305-0047 Japan; The Education Ministry Key Lab of Resource Chemistry, Shanghai Key Laboratory of Rare Earth Functional Materials, Shanghai Normal University Shanghai 200234 P. R. China; School of Chemical Engineering, Australian Institute for Bioengineering and Nanotechnology (AIBN), The University of Queensland Brisbane Queensland 4072 Australia y.yamauchi@uq.edu.au; Department of Plant and Environmental New Resources, Kyung Hee University 1732 Deogyeong-daero, Giheung-gu Yongin-si Gyeonggi-do 446-701 South Korea

## Abstract

Generating high surface area mesoporous transition metal boride is interesting because the incorporation of boron atoms generates lattice distortions that lead to the formation of amorphous metal boride with unique properties in catalysis. Here we report the first synthesis of mesoporous cobalt boron amorphous alloy colloidal particles using a soft template-directed assembly approach. Dual reducing agents are used to precisely control the chemical reduction process of mesoporous cobalt boron nanospheres. The Earth-abundance of cobalt boride combined with the high surface area and mesoporous nanoarchitecture enables solar-energy efficient photothermal conversion of CO_2_ into CO compared to non-porous cobalt boron alloys and commercial cobalt catalysts.

## Abstract 2

Generating high surface area mesoporous transition metal boride is challenging but interesting because incorporation of boron atoms can generate lattice distortion to form amorphous metal boride which has unique properties in catalysis.

## Graphical abstract

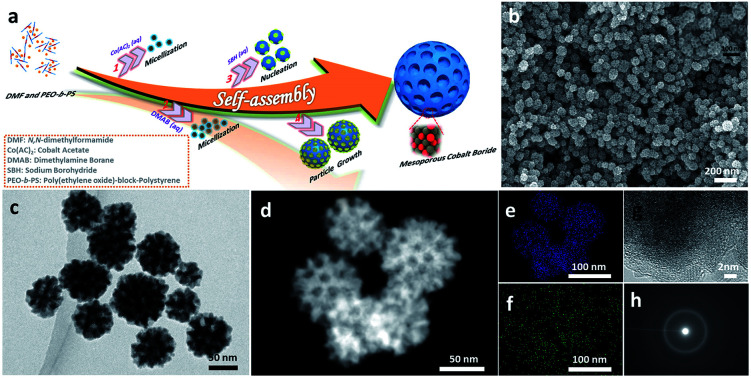

## Introduction

Colloidal metal nanoparticles have interesting electronic properties that enable a wide range of applications in heterogeneous catalysis, energy storage and conversion, fuel cells and chemical sensing.^1–5^ Generating even higher surface area metallic architectures increases the material utilization efficiency of the metal and exposes additional active sites for higher efficiency catalysis.^6–10^ But most reports on mesoporous metal colloids utilize precious metals such as Pt, Pd, and Ir and their alloys.^11–16^ There have been few examples of mesoporous architectures composed of nonprecious transition metals, despite their abundance in the Earth's crust. This is due in part to the sensitivity of transition metals to oxidation in air or solvents and their relatively low redox potential, which requires more strenuous reaction conditions and strong reducing agents.^17–19^ The combination of these factors makes the creation of high surface area mesoporous transition metals *via* soft-templated chemical synthesis very challenging despite all their potential advantages in terms of economic cost.

Alloying precious metals with transition metals is a common strategy to stabilize transition metals against oxidation and still access the unique electronic properties of the metal for catalysis. Recently, transition metal catalysts that incorporate boron (B) have gained a lot of attention because: (i) B can act as a donor or acceptor depending on metal concentration and help partially stabilize the transition metal against oxidation^20,21^ and (ii) B can generate lattice strain which affects the electronic structure of the metal and helps form lower-coordination unsaturated sites.^22–24^ Transition metal boride alloys and boron-doped metals exhibit enhanced activity/selectivity in versatile catalytic reactions such as the hydrogenation of cinnamaldehyde,^23^ electrochemical water splitting,^24^ electrocatalytic CO_2_ reduction,^25^ the oxygen reduction reaction,^26^ and the electro-oxidation of formic acid.^27^ For example, boron-doped Cu catalysts exhibit high selectivity for electrocatalytic reduction of CO_2_ into C_2_ products (Faraday efficiency ∼ 79%) because B modifies the local electronic structure of copper and creates positive valence sites.^22^ And amorphous cobalt boride (Co–B) has been reported as an exceptionally efficient catalyst for hydrogenation of cinnamaldehyde, due to the lattice strain that B generates in the crystal structure of the Co metal.^23^ These reports indicate that incorporation of boron into metals stabilizes a lot of the favorable electronic properties of transition metal catalysts in nanomaterial systems. Therefore, adapting these methods to generate mesoporous transition metal borides is a promising approach to access these kinds of catalytic properties in an ultra-high surface area system.

Here we describe a simple synthesis method to generate mesoporous amorphous cobalt boron (a-CoB_*x*_) alloy colloidal particles using block copolymer micelle templates as pore-directing agents. This method largely relies on the use of dual-reducing agents (*i.e.*, dimethylamine borane and sodium borohydride) to precisely control the reduction process and facilitate the incorporation of B into the metal. Sodium borohydride plays a key role in triggering the initially rapid nucleation of Co metal clusters/nanocrystals, while dimethylamine borane assists in the formation of the mesoporous structure as the Co metal is deposited around the micelle templates. Both sodium borohydride and dimethylamine borane serve as the B-source and form an amorphous cobalt boron alloy. The obtained mesoporous a-CoB_*x*_ alloys were then examined as catalysts for the photo-thermal conversion of CO_2_ with H_2_ to investigate the structure- and composition-dependent catalytic activity of the mesoporous network. The results show that mesoporous a-CoB_*x*_ nanoparticles have a higher performance toward photothermal assisted reduction of CO_2_ to CO *versus* non-porous a-CoB_*x*_ and commercial Co catalysts.

## Results and discussion

**Fig. 1:**
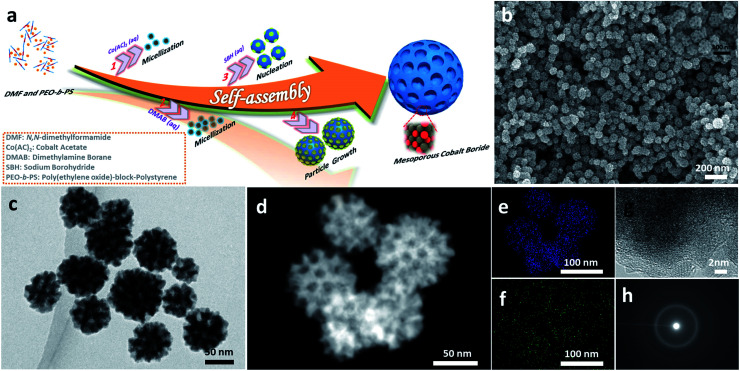
Illustration and microscopic characterization of mesoporous a-CoB_*x*_ alloy nanospheres. (a) An illustration describing the formation process. (b) SEM, (c) TEM, (d) HAADF-TEM, (e and f) elemental mapping, (g) HRTEM, and (h) SAED images of the as-prepared mesoporous a-CoB_*x*_ samples.

Fig. 1a illustrates the formation of mesoporous a-CoB_*x*_ nanoparticles using soft sacrificial pore-directing agents. In a typical synthesis, 10 mg of the block polymer PS-*b*-PEO was completely dissolved in an aprotic polar solvent *N*,*N*-dimethylformamide (DMF) (1.5 mL) as unimers because of the high solubility of both hydrophilic PEO and hydrophobic PS segments in DMF. Next an aqueous solution of 2.0 mL cobalt acetate (0.06 M) and 3 mL dimethylamine borane (0.5 M) was added into this solution, which drives the micellization of PS-*b*-PEO due to the low solubility of the PS segments in water. The optical signature of this phenomenon could be observed using the Tyndall effect (Fig. S1†). The resulting micelles were composed of PS cores surrounded by PEO shells with an average diameter of 12.7 nm, as shown in the TEM images using the phosphotungstic (PW) acid negative staining technique (Fig. S2†). This mixture in the flask was then degassed and purged under vacuum using a Schlenk line to remove dissolved oxygen from the solution and backfilled with the inert gas Ar to help protect the obtained samples from oxidization. The reaction took place immediately when a small amount of NaBH_4_ was added into the above solution. This solution was further incubated at room temperature for 1 h. Finally, the product was isolated by several consecutive washing/centrifugation cycles with acetone. Omitting the PS-*b*-PEO from the synthesis generated non-porous cobalt boron, indicating the essential role of the polymer as a pore-directing agent in the reaction (Fig. S3†).

Generally, the formation of mesoporous metals is accomplished through the cooperative assembly of reduced nanocrystals and micelles; learning how to chemically control the deposition process is a key point in the synthesis of mesoporous metals. NaBH_4_ is a commonly used reducing agent in the synthesis of all kinds of metal nanocrystals in aqueous systems. However, in most cases it is simply too strong a reducing agent to generate mesoporous structures and typically can only generate non-porous metals (Fig. S4a†). One can use weaker reducing agents like dimethylamine borane (DMAB), but they are not strong enough to reduce the Co metal precursor (Fig. S4b†). In this study, we examined how to combine NaBH_4_ and DMAB as dual-reducing agents to drive the reduction of Co while still generating the mesoporous structure (Fig. S5†).

The morphology and porous structure of the samples were characterized by scanning electron microscopy (SEM) and transmission electron microscopy (TEM). According to the SEM images, the as-prepared products are uniform in particle size with an average size of 70 ± 5 nm (Fig. 1b and S6a–c†) and the well-defined mesopores are clearly distributed over the entire outer surface of the nanoparticles (inset Fig. 1b). The pore size and the thickness of the pore wall were estimated to be 11.6 ± 1 nm and 10.4 ± 1 nm, respectively (Fig. S6d and e†), from the SEM images. The internal structure of nanoparticles was further studied by TEM, which showed that the mesopores were present in the interior of the nanoparticles as well (Fig. 1c and d). The energy dispersive X-ray elemental mapping images (Fig. 1e and f) confirm that Co and B are uniformly distributed throughout the nanoparticles. The molar ratio of cobalt to boron is 5.05 : 1.00, according to inductively coupled plasma optical emission spectroscopy (ICP-OES) (the boron concentration is around 3.5 wt%). The local crystal structure of the nanoparticles was analyzed with high-resolution TEM (HRTEM) and selected area electron diffraction (SAED) (Fig. 1g and h). The HRTEM image shows that the as-prepared samples do not show any clear lattice fringes, although a very short-range ordered atomic arrangement is found in a few locations (Fig. S7†). Intense dots are not observed in the SAED patterns, strongly indicating that the structure is amorphous, which is a feature of cobalt boron alloys.^23,28^

**Fig. 2:**
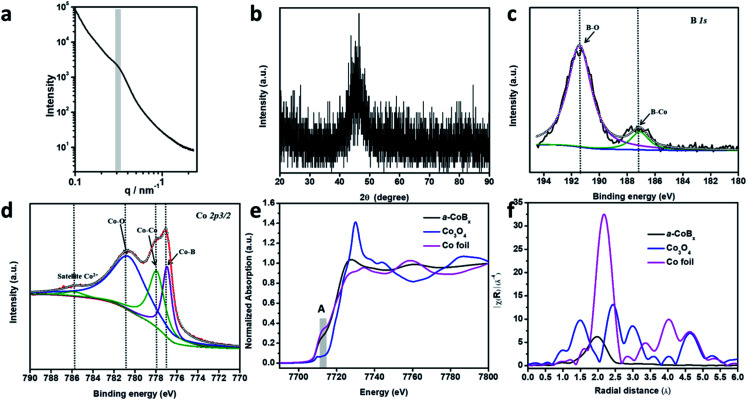
Detailed structural characterization of the a-CoB_*x*_. (a) Small angle X-ray scattering pattern and (b) wide-angle XRD pattern of mesoporous a-CoB_*x*_ samples. (c) XPS spectrum of the B 1s peak and (d) the Co 2p_3/2_ peak in the a-CoB_*x*_ sample. (e) XANES and (f) EXAFS spectra of the cobalt K-edge in a-CoB_*x*_ and in the references Co foil and Co_3_O_4_.

In order to study the periodicity of the mesoporous structure, small angle X-ray scattering (SAXS) measurements were carried out, as shown in Fig. 2a. A broad peak centered at 0.31 nm^−1^ (*i.e.*, pore-to-pore distance of 20.3 nm) is clearly observed in the SAXS profile, demonstrating that uniformly sized spherical pores are closely packed with each other. The Brunauer–Emmett–Teller (BET) surface area of mesoporous a-CoB_*x*_ is determined to be 44.2 m^2^ g^−1^, which is around 5 times and 10 times higher than that of non-porous a-CoB_*x*_ (7.8 m^2^ g^−1^) and commercial Co (4.5 m^2^ g^−1^), respectively. The crystalline phase of the obtained sample was confirmed by wide-angle XRD (Fig. 2b). It has one broad, low intensity peak at ∼45°, generally caused by incorporation of boron atoms.^20,29^ Next, X-ray photoelectron spectroscopy (XPS) was used to study the surface electronic structure of Co and B. The high resolution B 1s spectrum can be deconvoluted into two distinct peaks with binding energies of 187.1 and 191.3 eV, showing that there are both Co–B species and oxidized borate, respectively (Fig. 2c).^30,31^ The Co 2p_3/2_ spectrum in Fig. 2d has a peak at 778.0 eV which matches Co^0^ in metallic Co.^32^ In addition, the peak with a low binding energy of 777.0 eV in Fig. 2d can be assigned to the interaction of Co^0^ with boron caused by reverse electron-transfer from the B to the Co atom.^20,33^ Co^2+^ species (*i.e.* CoO or Co(OH)_2_) are detected in the as-prepared sample as well as in commercial Co (Fig. 2d and S8†), indicating that spontaneous oxidation of Co or a-CoB_*x*_ surfaces occurs in both cases.^34,35^

The local structural environment of the Co element in the mesoporous a-CoB_*x*_ catalyst was further investigated by X-ray absorption spectroscopy (XAS) measurements. The cobalt K-edge is frequently used as a qualitative fingerprint to identify cobalt species.^36^ In the X-ray absorption near edge structure (XANES) spectrum, a-CoB_*x*_ has a pre-edge peak (A) with a position and shape that matches the Co foil reference material (Fig. 2e). The first feature above the absorption edge (white line) indicates the presence of some oxidized cobalt, which is in agreement with the XPS observation. Extended X-ray absorption fine structure (EXAFS) data show that the a-CoB_*x*_ sample has a single peak at a radial distance of 1.96 Å, which is assigned to the Co–Co scattering pair in a-CoB_*x*_, smaller than that of Co–Co (2.1 Å) in the metal foil standard sample (Fig. 2f). This indicates that boron induces some stain in the crystal lattice of cobalt, likely resulting from electron transfer and hybridization of B 2p states with metal d orbitals.^24,37^ The reduced intensity beyond the first shell peak is ostensibly caused by incorporation of B atoms around Co center atoms, generating a more local disordered structure.^23^ This may also generate a higher concentration of unsaturated metal sites in the a-CoB_*x*_ sample (Table S1†), which is conducive for higher activity in catalytic reactions.

**Fig. 3:**
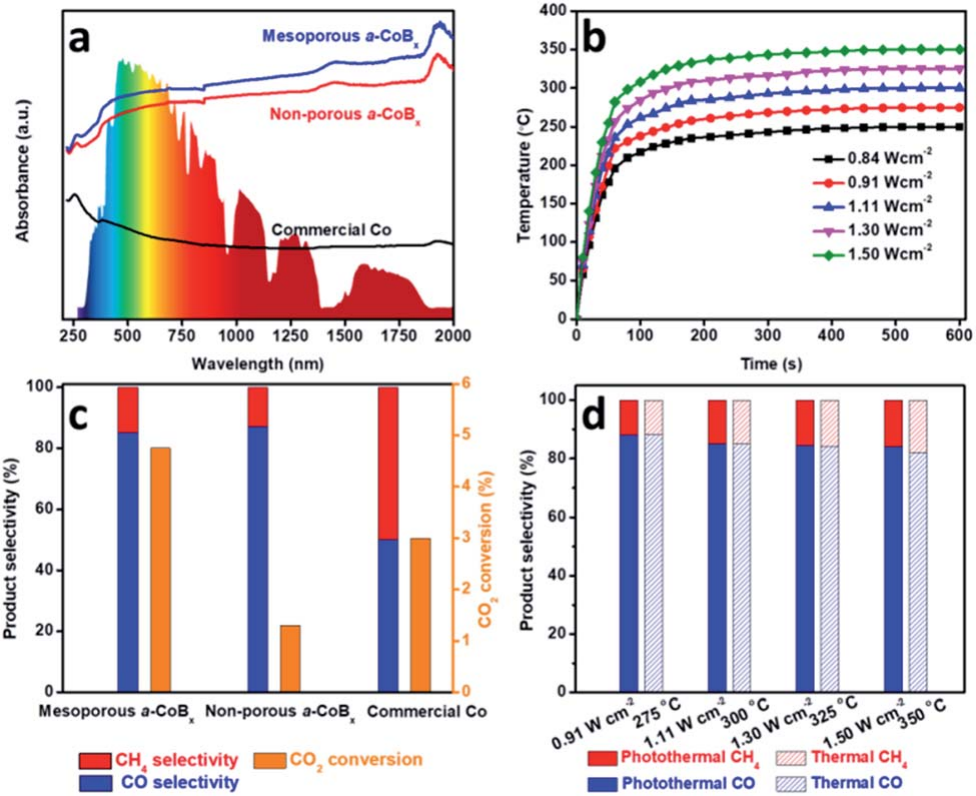
Catalytic performances of the a-CoB_*x*_ for photothermal CO_2_ conversion. (a) UV/Vis/NIR spectrum of the mesoporous a-CoB_*x*_, non-porous a-CoB_*x*_ and commercial Co. The inset in (a) shows a solar irradiance spectrum. (b) The required irradiation light intensities over mesoporous a-CoB_*x*_ materials to reach the target temperature for driving the catalysis, (c) activity and selectivity of the mesoporous a-CoB_*x*_, non-porous a-CoB_*x*_ and commercial Co catalysts, and (d) comparison of selectivity over the mesoporous a-CoB_*x*_ catalyst under photothermal (light irradiation) and direct thermal heating (no irradiation). For the photothermal (light irradiation) system, we applied 0.91, 1.11, 1.30, and 1.50 W cm^−2^, respectively, to achieve the target temperatures.

Photo-thermal catalysis is a low-energy input renewable technology using solar energy to generate heat to help drive catalytic reactions.^38–43^ Nanostructured metals are good candidate materials for photo-thermal catalysis because they have high thermal conductivities, strong absorption cross-sections, and intrinsic catalytic properties. We evaluated the performance of mesoporous a-CoB_*x*_, non-porous a-CoB_*x*_ and commercial Co catalysts (Fig. S9†) in the photo-thermal hydrogenation of CO_2_ in a flow-type reactor. The structural thermostabilities were initially examined by annealing a typical sample (*i.e.*, mesoporous a-CoB_*x*_) at different temperatures (*e.g.*, 300, 325, and 350 °C) for 1 h in N_2_ (Fig. S10a–c†). There is no obvious change in the mesoporous structure or particle aggregation as observed by SEM even after the thermal treatment at 350 °C. The wide-angle XRD patterns show the amorphous features of each of the annealed samples (Fig. S10d†). These results indicate that mesoporous a-CoB_*x*_ nanoparticles are stable enough to maintain their structural features even at the high temperatures used in photo-thermal catalytic reactions. Then UV-Vis-NIR absorption properties of mesoporous a-CoB_*x*_, non-porous a-CoB_*x*_ and commercial Co were characterized in a reflectance setup. They show that the a-CoB_*x*_ particles strongly absorb light, even in the NIR region (Fig. 3a), indicating that they have a high capacity for photo-to-thermal conversion. The efficient photo-to-thermal conversion by mesoporous a-CoB_*x*_ is probably attributed to the synergistic effect of B-doping and multi-reflections of incident light within the mesoporous structure.^44,45^ The relationship between the target temperature and light intensity over various catalysts was investigated using a 300 W Xe lamp equipped with an optical lens as the light source (Fig. 3b, S11a and b†) and is summarized in Fig. S11c†. At a light intensity of 1.50 W cm^−2^, the measured temperature over mesoporous a-CoB_*x*_ increases rapidly and reaches 350 °C within five minutes, whereas the temperatures over non-porous a-CoB_*x*_ and commercial Co catalysts are 312 and 290 °C, respectively (Fig. S11d†).

To evaluate their performance, the obtained mesoporous catalysts were investigated for CO_2_ hydrogenation at atmospheric pressure using a H_2_/CO_2_ ratio of 4/1. CO and CH_4_ were the only detectable reaction products. Since CH_4_ is a greenhouse gas, controlling the hydrogenation of CO_2_ to produce carbon monoxide (CO), an valuable feedstock, is highly desired. The selectivity of CO_2_ conversion toward CO *versus* CH_4_ was measured at a light intensity of 1.30 W cm^−2^, 1.59 W cm^−2^ and 1.72 W cm^−2^ for mesoporous a-CoB_*x*_, non-porous a-CoB_*x*_, and commercial Co, respectively (*i.e.*, the catalyst temperature was 325 °C). The mesoporous a-CoB_*x*_ catalyst is able to more efficiently convert CO_2_ compared to non-porous a-CoB_*x*_ and commercial Co (Fig. 3c). The higher efficiency of our mesoporous a-CoB_*x*_ for CO_2_ conversion can be attributed to numerous factors related to the mesoporous structure including higher surface area and more active sites in the amorphous alloy. The a-CoB_*x*_ samples (*i.e.*, mesoporous and non-porous a-CoB_*x*_) exhibit higher CO selectivity (Fig. 3c). Density functional theory (DFT) based on the Co cluster models indicates that the decreased CO adsorption energy on the surface of the CoB catalyst (Fig. S12 and Table S2†) is responsible for achieving high CO selectivity. The light intensity-dependent CO selectivity and CO_2_ conversion were also investigated by modulating the intensity of the excitation source (Fig. 3d). This shows that selectivity is largely unchanged with the increasing temperature; however, the CO_2_ conversion efficiency is expected to increase with higher light intensities (Fig. S13†). The CO selectivity and CO_2_ conversion efficiency are roughly the same under photo-irradiation heating *versus* direct external heat (*i.e.*, without light irradiation) over the entire range tested, as shown in Fig. 3d and S13.† This indicates that the reaction is driven by photo-thermal catalysis rather than photocatalysis. Finally, the mesoporous a-CoB catalyst displays excellent material stability in both conversion efficiency and CO selectivity (Fig. S14†) over 6 h under photo-irradiation with a light intensity of 1.11 W cm^−2^ and shows no noticeable change in morphology (Fig. S15†).

## Conclusions

In summary, mesoporous non-precious metal cobalt boron alloy nanoparticles have been synthesized for the first time using a simple chemical reduction approach in the presence of polymeric micelle pore-directing agents. The precise control of the deposition process is responsible for the formation of the mesoporous structure. Due to the ultra-high surface area with highly unsaturated active sites in the amorphous structures and the efficient photo-thermal conversion of the catalyst, high CO_2_ conversion efficiencies can be successfully realized. The incorporation of boron causes lattice strain in the metal, reducing the CO adsorption energy for high CO selectivity compared with commercial Co. This work opens new avenues for developing non-precious mesoporous metal boride alloys and enables new prospects for the use of metal borides in light-enhanced photothermal reactions.

## Conflicts of interest

There are no conflicts to declare.

## Supplementary Material

SC-011-C9SC04498A-s001
