# Supplementary material for: A mesoporous non-precious metal boride system: synthesis of mesoporous cobalt boride by strictly controlled chemical reduction
Source: Chem Sci. 2019 Nov 15;11(3):791–6. doi: 10.1039/c9sc04498a (PMC8145993; doi:10.1039/c9sc04498a)
Supplement: SC-011-C9SC04498A-s001 [file SC-011-C9SC04498A-s001.pdf]

Electronic Supplementary Material (ESI) for Chemical Science.
This journal is © The Royal Society of Chemistry 2019

Materials and Methods

Materials.

Cobalt acetate, dimethylamine borane, sodium borohydride, were purchased from Sigma-Aldrich Chemical Co. Poly(ethylene oxide)-*b*-polystyrene ($PEO_{(2200)}$-*b*-$PS_{(5000)}$) was purchased from Polymer Source Inc. *N,N*-dimethylformamide, and acetone were purchased from Nacalai Tesque, Inc. All the chemicals were used without further purification.

Preparation of mesoporous *a*-CoB_x nanoparticles.

The synthesis of mesoporous *a*-CoB_x nanoparticles is based on a wet chemical reduction process in the solution phase. In a typical synthesis, 10 mg block polymer PS-*b*-PEO was completely dissolved in an aprotic polar solvent *N,N*-dimethylformamide (DMF) (1.5 mL). Next an aqueous solution of 2.0 mL cobalt acetate (0.06 M) and 3 mL dimethylamine borane (0.5 M) was added to this solution. This mixture in the flask was then degassed and purged under vacuum using a Schlenk line to remove dissolved oxygen from the solution, backfilled with inert gas Ar to help protect the cobalt metal from oxidization. The reaction took place immediately when small amount of $NaBH_4$ (200 μL, 5 $mg \cdot mL^{-1}$) was added into above solution. This solution was further incubated at room temperature for 1 h. Finally, the product was isolated by several consecutive washing/centrifugation cycles with acetone.

Solar light-driven CO_2 hydrogenation reaction.

The solar light-driven CO_2 hydrogenation reaction was performed at atmospheric pressure in a homemade flow-type reactor equipped with a quartz window to allow light irradiation. 300 W Xe lamp equipped with an optical lens was used as a light source. The irradiation from the lamp was focused by the optical lens into the reactor. In general, 10 mg of catalyst was dispersed in the reaction cell. The react cell was flushed with pure Ar gas for 2 h to remove the air in the cell. Then, the mesoporous *a*-CoB_x and commercial Co catalysts were firstly pretreated for 1 h to react stable catalytic performance at light intensities of 0.91 W cm^{-2} and 1.33 W cm^{-2}, respectively, in a mixture gas of CO_2 (20%) and H_2 (80%) with a flow rate of 10 mL min^{-1}. All products were detected and quantified by a gas chromatograph (GC) equipped with flame ionization detector. The light intensity was adjusted by tuning the current of Xe lamp and was detected by USR-40 spectrophotometer. The temperatures of all materials were determined by digital thermometer (CT1200D)

Thermocatalytic CO_2 hydrogenation reaction.

The thermocatalytic CO_2 hydrogenation reactions at different temperatures were conducted at atmospheric pressure in a flow-type reactor. In general, 10 mg of catalyst was dispersed in the reaction cell. Then, a mixture gas of CO_2 (20%) and H_2 (80%) with a flow rate of 10 mL min^{-1} was introduced into the reactor. A thermocouple was contacted with the catalyst sample to detect the reaction temperature. All products were quantified by gas chromatograph (GC) equipped with flame ionization detector.

Characterization.

Field emission scanning electron microscope (SEM, HITACHI SU-8000) was used to observe the morphology of mesoporous cobalt boron nanoparticles with the accelerating voltage of 5 kV. High resolution transmission electron microscopy (HRTEM, JEOL JEM-2100F) operated at 200 kV was used to investigate the structure of the mesoporous nanoparticles. The samples for TEM and HRTEM measurements were prepared by depositing a drop of the diluted colloidal suspension on a TEM grid. Powder X-ray diffraction (XRD) measurements were conducted on a Smart lab X-ray diffractometer (RIGAKU) at a scanning rate of 1 deg min^{-1} with a Cu Kα radiation (40 kV, 30 mA) source. Small angle X-ray scattering (SAXS) measurements (Rigaku NANO-Viewer) were used to evaluate the pore-to-pore distance. X-ray photoelectron spectroscopy (XPS) was performed on a JPS-9010TR (JEOL) instrument with an Mg Kα X-ray source. All the binding energies were calibrated using the C 1s binding energy peak (284.5 eV) as a reference. Inductively coupled plasma optical emission spectrometer (ICP-OES) was performed on a Hitachi model SPS3520UV-DD. Nitrogen adsorption-desorption isotherms were acquired by using a BELSORP-mini (BEL, Japan) at 77 K and surface area was estimated using the Multipoint Brunauer-Emmett-Teller (BET) method. The UV-Vis-IR optical properties of the samples were investigated on UV/vis/NIR-spectrometer V-570 (Jasco Inc.) from 200 to 2000 nm.

Co K-edge X-ray absorption spectra were acquired at beamline 1W1B of the Beijing Synchrotron Radiation Facility (BSRF) in fluorescence mode at room temperature using a Si (111) double-crystal monochromator. The storage ring of BSRF was operated at 2.5 GeV with a maximum current of 250 mA in decay mode. The energy was calibrated using Co foil; the intensities of the incident and fluorescence X-ray were monitored by using standard N_2-filled ion chamber and Argon-filled Lytle-type detector, respectively. In order to achieve the best signal-to-noise ratio, the powdered samples were uniformly mixed with BN

powder and pressed to a pellet, which was sealed in a cell holder with Kapton windows for XAFS measurement. A detuning of about 20 % by misaligning the silicon crystals was also performed to suppress the high harmonic content. The XAFS raw data were background-subtracted, normalized, and Fourier transformed by the standard procedures with the ATHENA program. To obtain the quantitative structural parameters around Co atoms in our samples, the least-squares curve parameter fitting was performed using the ARTEMIS module of IFEFFIT software packages. The amplitude reduction factor S02 was treated as adjustable variable and the obtained value of 0.85 for Co-foil was fixed in fitting the subsequent Co edge data for the samples. The fit was performed on the k3-weighted EXAFS function $\chi(k)$ data from 3 to 12.3 Å-1 in the R-range of 1.0-3.0 Å. The coordination numbers N, interatomic distances R, Debye-Waller factor σ2 and the edge-energy shift ΔE0 for were allowed to run freely.

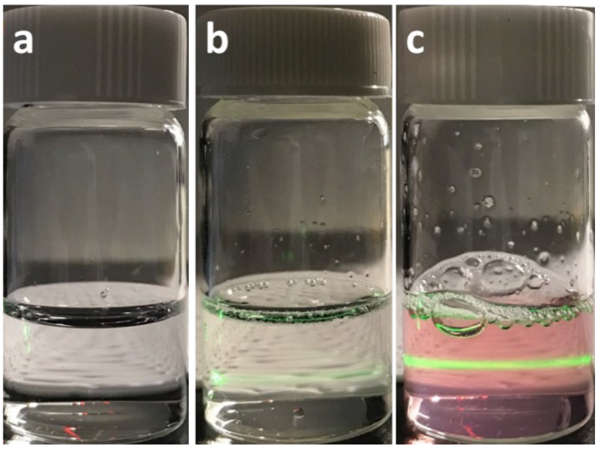

Fig. S1 Optical micrographs demonstrating the emergence of the Tyndall effect in the solution with different compositions (a) PEO-*b*-PS dissolved in DMF, (b) PEO-*b*-PS dissolved in DMF and water, and (c) PEO-*b*-PS dissolved in DMF + water + Co precursor + DMAB.

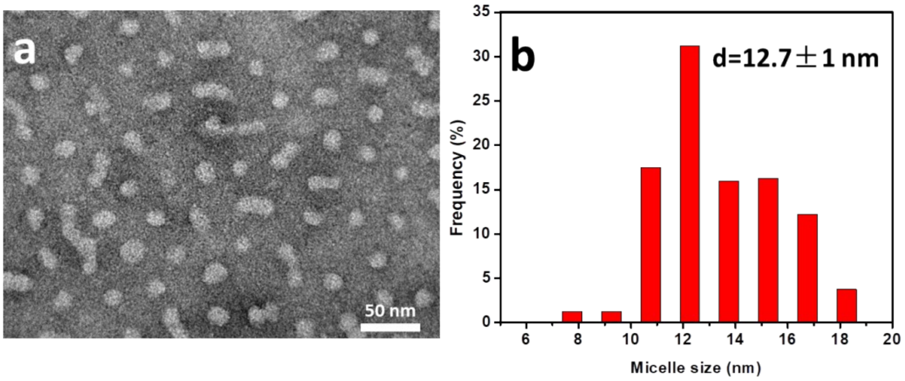

Fig. S2 (a) TEM images and (b) histograms of particle size distribution of the polymeric micelles obtained from mixture solution of DMF + PEO-*b*-PS + water

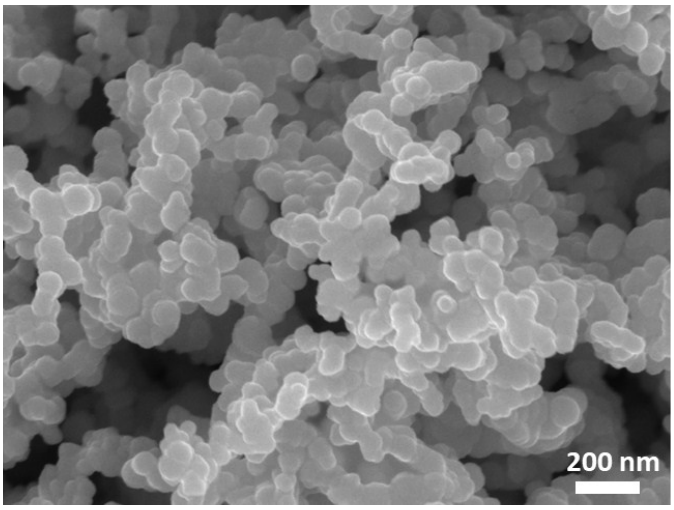

Fig. S3 SEM image of *a*-CoB_x samples prepared in the absence of polymer.

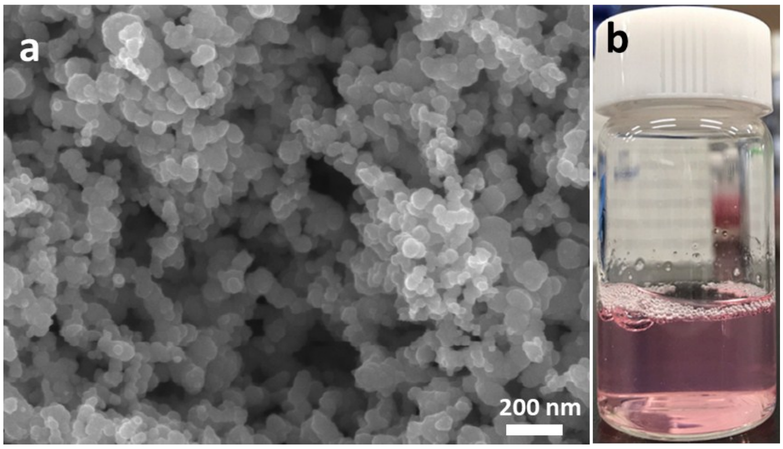

Fig. S4 (a) SEM image of the *a*-CoB_x prepared by using the $NaBH_4$ as a reducing agent, (b) the reaction solution after adding DMAB for 1 h.

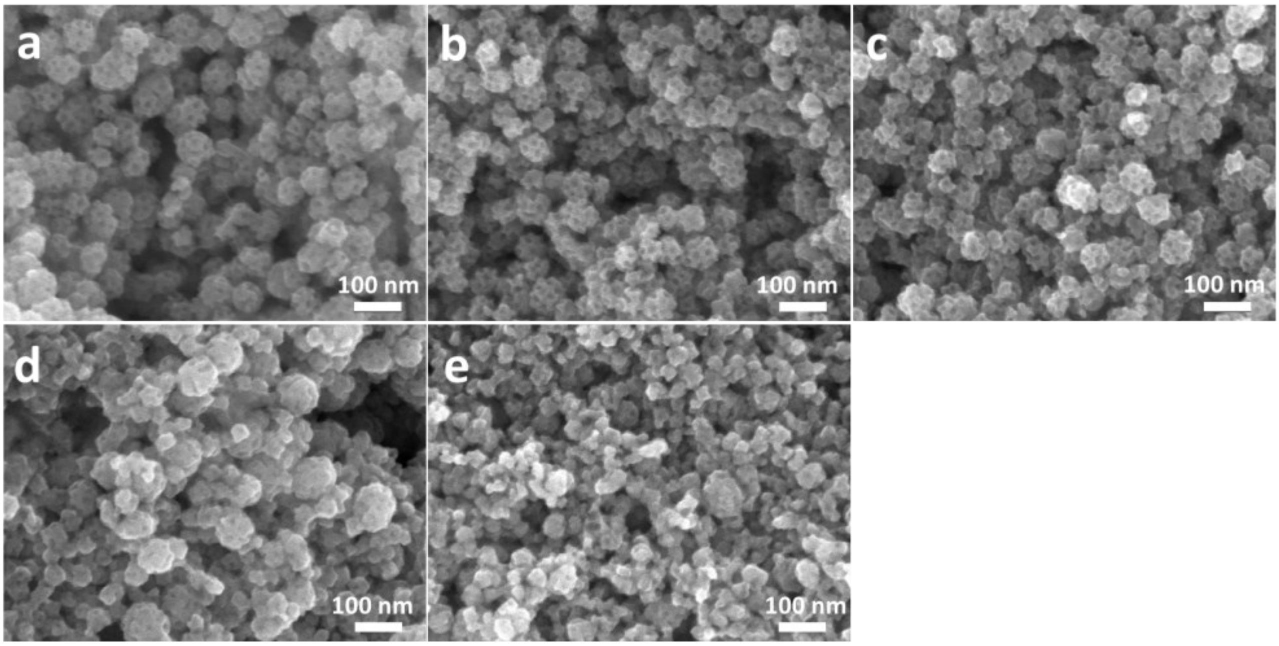

Fig. S5 SEM images of as-prepared samples using different volume of $NaBH_4$ solution (5 mg·mL^{-1}) in the presence of polymer ((a) 50 μL, (b) 200 μL, (c) 500 μL, (d) 1000 μL, and (e) 2000 μL, respectively.).

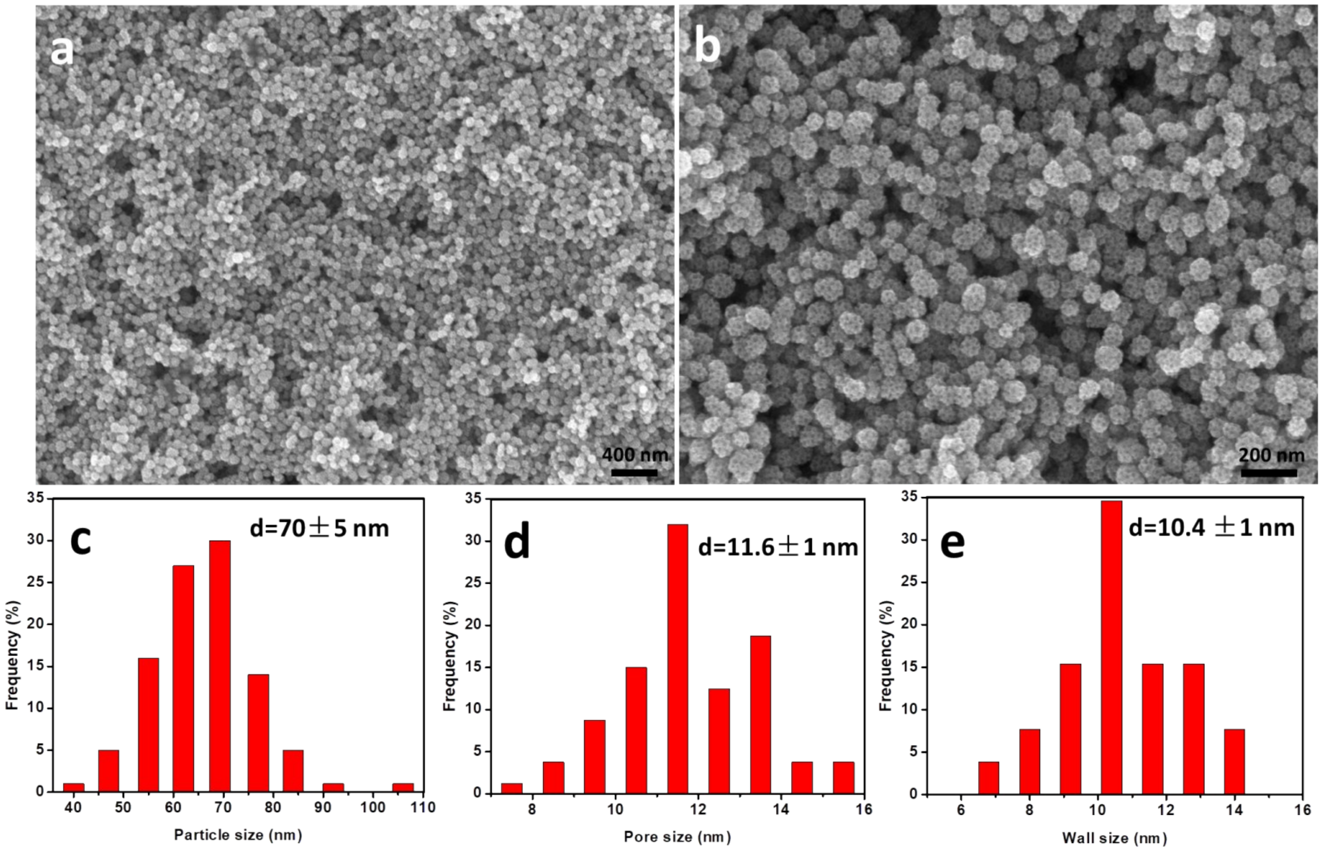

Fig. S6 (a,b) SEM image of the typical mesoporous *a*-CoB_x samples, (c) histograms of particle size distribution, (d) pore size distribution, and (e) thickness of the pore wall of the mesoporous *a*-CoB_x sample.

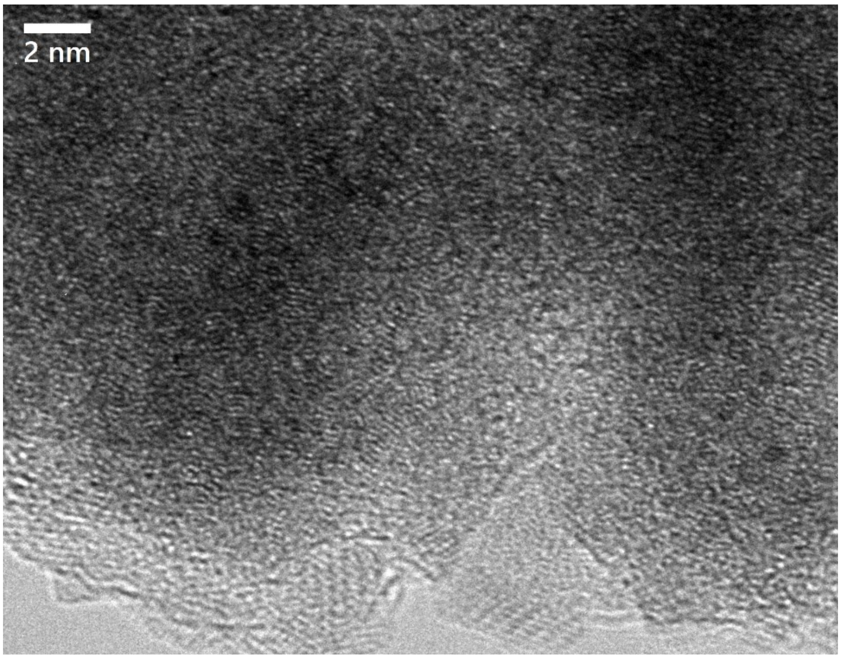

Fig. S7 High resolution TEM image of the pore wall of the mesoporous *a*-CoB_x sample.

Table S1 Co K-edge EXAFS fitting of mesoporous *a*-CoB_x and Co foil

Sample	Scattering path	CN	R(Å)	$\sigma^2(10^{-3}Å^{-2})$	ΔE_0(eV)
a-CoB_x	Co-B	1.84	2.05	5.8	5.6
	Co-Co	2.11	2.20	10.5	29.7
Co-foil	Co-Co	12	2.49	7.1	8.2

Note: CN: coordination number, R: scattering distance, σ^2 = EXAFS Debye-Waller factor, ΔE = atomic potential shift.

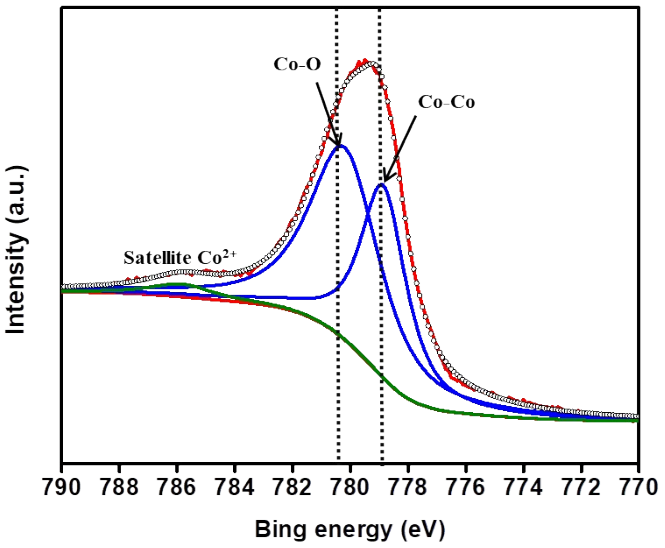

Fig. S8 XPS spectrum of the Co $2p_{3/2}$ peak in commercial Co.

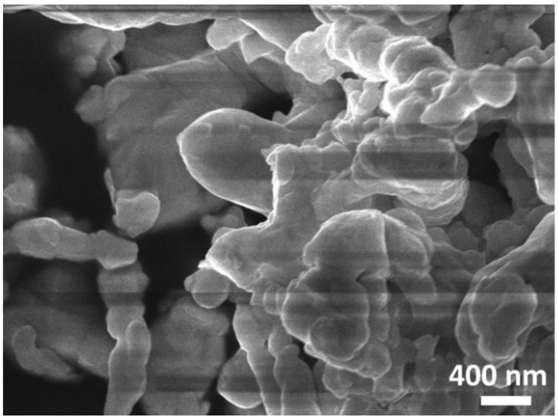

Fig. S9 SEM image of the commercial Co sample.

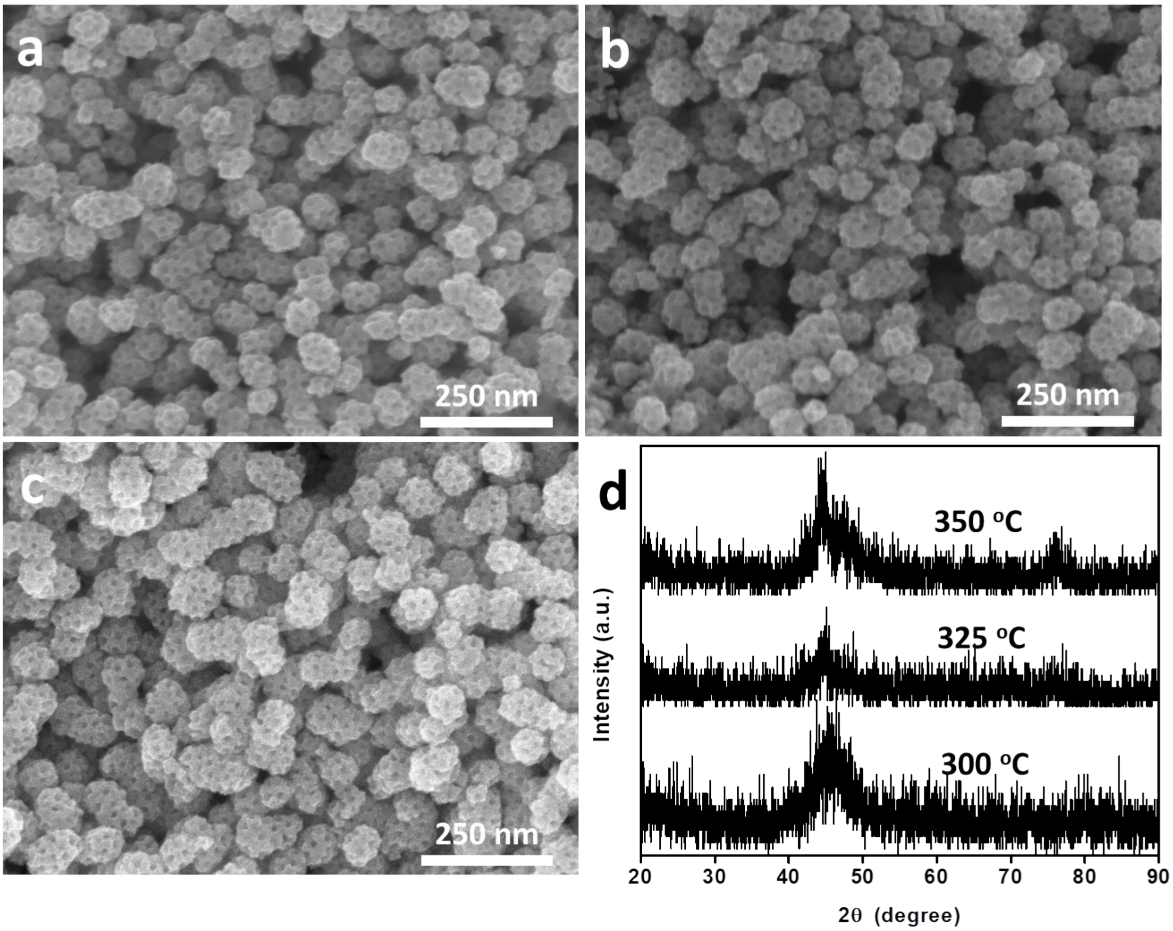

Fig. S10 SEM images of the mesoporous *a*-CoB_x nanoparticles annealed at 300 (a), 325 (b), and 350 °C (c), and (d) wide-angle XRD of the mesoporous *a*-CoB_x nanoparticles.

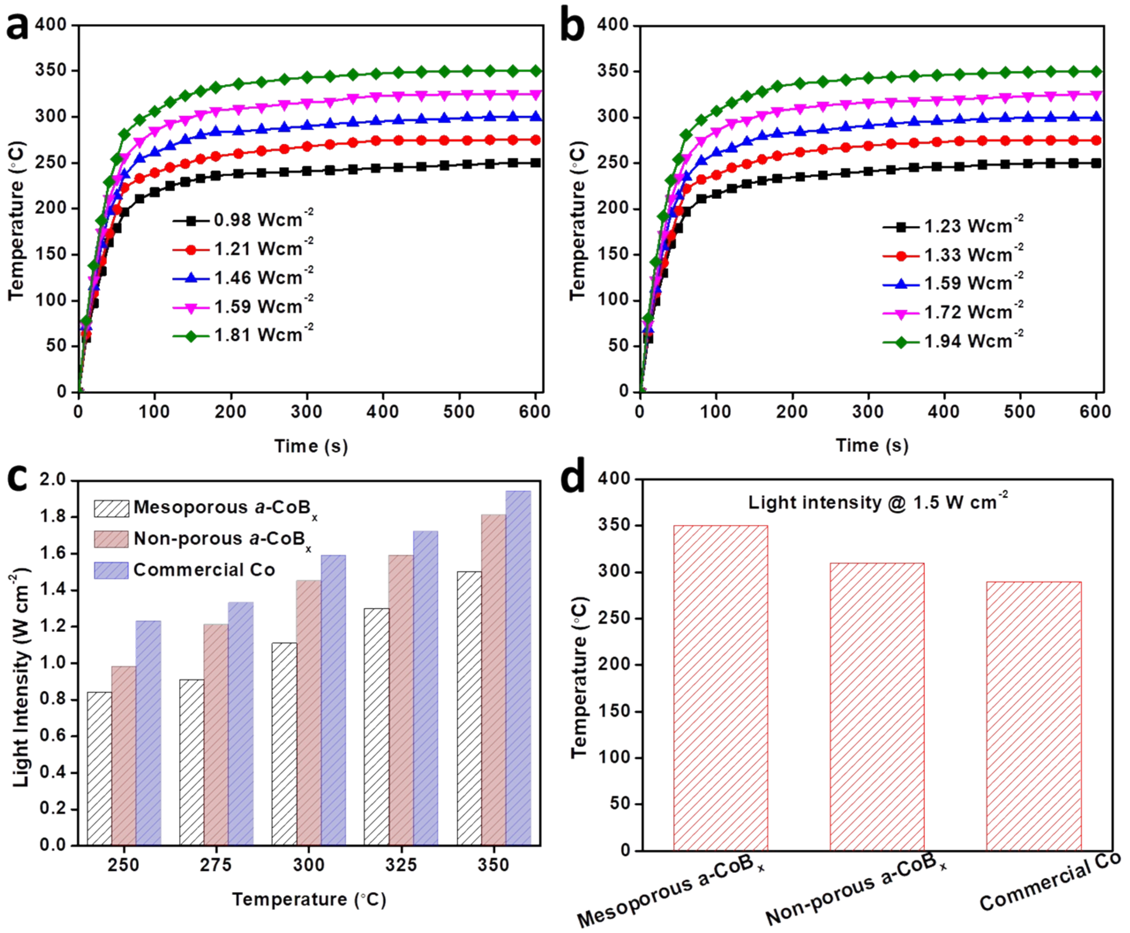

Fig. S11 (a,b) The relationship between the increased temperature and light intensities for non-porous *a*-CoB_x, and commercial Co materials, respectively. (c) Summary of the relationships between the target temperature and the light intensity of each sample. (d) The corresponding temperature of mesoporous *a*-CoB_x, non-porous *a*-CoB_x and commercial Co catalysts at the light intensity of 1.5 Wcm^{-2}.

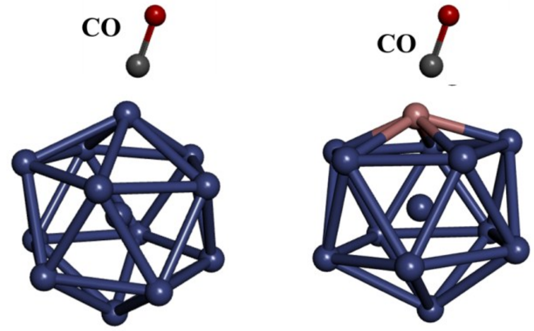

Fig. S12 Cluster models of Co_{13} and $Co_{12}B$, respectively.

Table S2 Calculated adsorption energies of CO adsorbed on Co and *a*-CoB clusters.

Co_{13}	-3.647874
$Co_{12}B$	-2.425965
	Absorption Energy

Computational details: Our first-principle calculation was based on density functional theory (DFT) and performed in the SIESTA (Spanish Initiative for the Electronic Simulations of Thousand of Atoms) program package.[1-3] In this calculation, the double zeta basis set plus polarization (DZP) function was used for all elements. The exchange and correlation potential was used in the generalized gradient approximation (GGA) in the scheme of Perdew, Burke and Ernzerrof (PBE).[4] All the geometry structures were relaxed until energy and force are converged to 10^{-4} eV and 0.02 eV/Å. A cutoff of 130 Ry was used for the real-space integration grid. Brillouin zone was sampled by 3×3×3 k-points grid for optimize. In this calculation, the lattice constant of supercells was 10×10×10. To investigate the behavior of CO absorption on the Co cluster, the absorption energy Eads was put forward and defined as where Eads Etotal (Ecluster ECO) where Eads was the total energy of adsorbate (*i.e.* CO) on Co cluster, Ecluster and ECO were the total energy of cluster and adsorbate, respectively. In this condition, the more negative value of Eads stood for more stable adsorption.

References

[1] P. Ordejo´n, E. Artacho, J. Soler, *Phys. Rev. B: Condens. Matter Mater. Phys*. **1996**, 53, R10441;

[2] D. Sa´nchez-Portal, P. Ordejo´n, E. Artacho, J. M. Soler, *Int. J. Quantum Chem.* **1997**, 65, 453;

[3] J. M. Soler, E. Artacho, J. D. Gale, A. Garcı´a, J. Junquera, P. Ordejo´n, D. Sa´nchezPortal, *J. Phys. Condens. Matter* **2002**, 14, 2745.

[4] J. P. Perdew, K. Burke, M. Ernzerhof, *Phys. Rev. Lett.* **1996**, 77, 3865

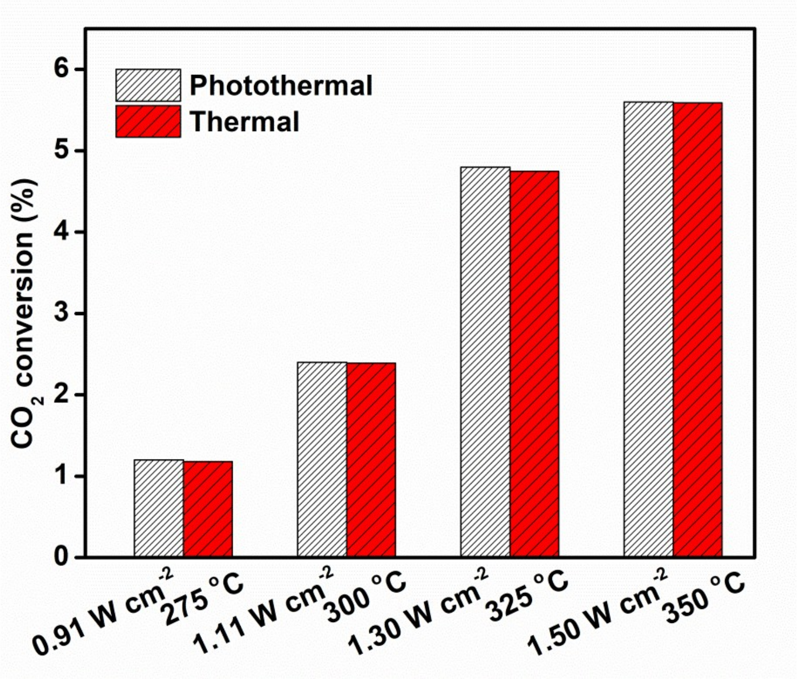

Fig. S13 Comparison of conversion over mesoporous *a*-CoB_x catalyst under photothermal (light irradiation) and direct thermal heating (no irradiation). For the photothermal (light irradiation) system, we applied 0.91, 1.11, 1.30, and 1.50 W cm^{-2}, respectively, to achieve the target temperatures.

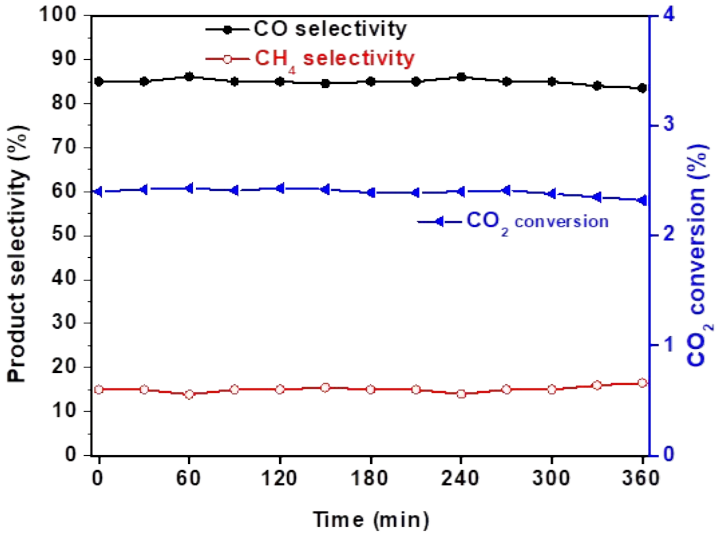

Fig. S14 Stability test of mesoporous *a*-CoB_x catalyst at light intensity of 1.11 Wcm^{-2}.

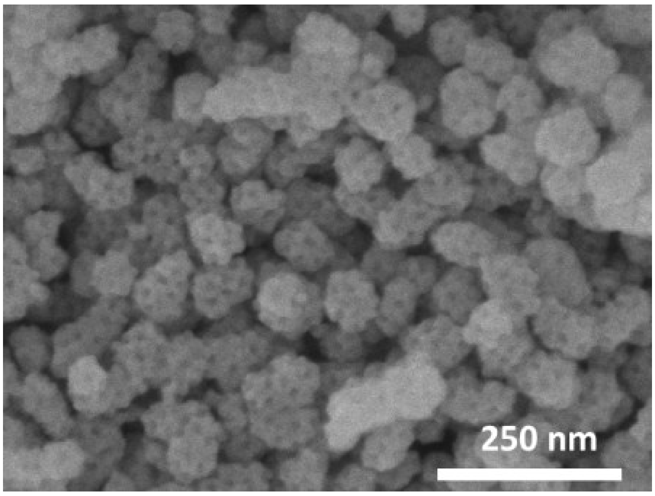

Fig. S15 SEM image of the mesoporous *a*-CoB_x after stability test at light intensity of 1.11 Wcm^{-2}.
